# Methods of Evaluating Large Language Model–Based Health Care Applications Used by Nonprofessionals: Protocol for a Scoping Review

**DOI:** 10.2196/93509

**Published:** 2026-08-11

**Authors:** Maren Keuchel, Pinar Bisgin, Tom Strube, Niklas Tschorn, Leoni Weltermann, Sven Meister

**Affiliations:** 1 Health Care Informatics, Faculty of Health School of Medicine Witten/Herdecke University Witten, North Rhine-Westphalia Germany; 2 Department of Healthcare Fraunhofer Institute for Software and Systems Engineering Dortmund, North Rhine-Westphalia Germany

**Keywords:** large language model, LLM, evaluation, health care, quality, artificial intelligence, AI

## Abstract

**Background:**

Large language models (LLMs) are increasingly used in health care by nonprofessionals (ie, individuals without formal training in health-related professions). These applications must be evaluated in an appropriate manner to prevent misinformation and harmful decisions. To date, guidance to evaluate LLM-based applications for nonprofessional users remains limited and fragmented, leaving researchers and developers without a scientifically grounded set of quality dimensions, metrics, and measurement tools to guide them.

**Objective:**

This protocol outlines a scoping review that maps approaches for evaluation of LLM-based applications used for health purposes by nonprofessionals. It identifies current methods and maps them thematically by assigning them to evaluation dimensions, metrics, and measurement instruments. The review will provide a comprehensive overview of evaluation methods currently in use.

**Methods:**

The study follows the Joana Briggs Institute approach for conducting scoping reviews and reports. The protocol is reported in accordance with the PRISMA-P (Preferred Reporting Items for Systematic Reviews and Meta-Analyses Protocols) guidelines, and the scoping review will be reported in accordance with the PRISMA-ScR (Preferred Reporting Items for Systematic Reviews and Meta-Analyses extension for Scoping Reviews) guidelines. The inclusion criteria comprise studies that evaluate LLM-based applications that are used in the context of health care by nonprofessionals. The search was conducted in PubMed, CINAHL, PsycInfo, and IEEE Xplore. Results since 2021 were considered. Data will be summarized and interpreted qualitatively. Publication screening was conducted by 2 independent reviewers in a blinded manner, with discrepancies settled through discussion. Data extraction and charting will be performed by 1 reviewer. To ensure quality, a random 10% sample of the publications will be independently charted by a second reviewer. Disagreements in the double-extracted subset will be resolved through discussion.

**Results:**

As of July 2026, a steering committee of 6 researchers has been chosen for the conduct of the review. An initial search resulted in 8538 records after removing duplicates. After screening of these 8538 publications, 17.8% (1524/8538) were eligible for retrieval, of which 88.3% (1345/1524) were retrieved. Full-text screening (completed by 1 reviewer) excluded publications due to nonmatching populations (155/1345, 11.5%), concepts (246/1345, 18.3%), and contexts (24/1345, 1.8%), as well as secondary work (14/1345, 1%), leaving 67.4% (906/1345) of these publications for data extraction. We plan to perform final full-text screening, data extraction, coding, and synthesis of results in the fourth quarter of 2026.

**Conclusions:**

The scoping review aims to identify and map current evaluation methods for LLM-based applications used in health care by nonprofessionals. It will provide a systematic overview of the current state of research and insights into quality dimensions, metrics, and measurement instruments. The findings will provide directional guidance for further research and development in the field of quality assurance for LLM-based applications used by nonprofessionals.

**International Registered Report Identifier (IRRID):**

DERR1-10.2196/93509

## Introduction

### Background

Large language model (LLM)–based health care applications are software systems that use LLMs to perform tasks that maintain and improve the health of populations and individuals [1]. The number of LLM-based applications in the field of health care is rapidly increasing [2]. They are being deployed across a diverse range of domains and use cases, such as chatbots answering medical questions, generation of patient information, clinical documentation, translation and summarization, and the creation of patient education material [3,4]. Health care professionals such as physicians, nurses, or pharmacists use LLM-based applications mainly to support clinical decision-making [5]. In this context, nonprofessionals are individuals that lack formal education with theoretical and factual knowledge on the diagnosis and treatment of health problems [6]. They use LLM-based applications mainly to educate themselves on medical topics; interpret medical information; and receive lifestyle recommendations, support in customized medication use, perioperative care instructions, and support in physician-patient interaction [7]. LLM-based applications offer promising solutions to many challenges faced by nonprofessionals. However, as this is still a relatively new field of research, there is also a high risk associated with their use in health care, especially when used by nonprofessionals as they may not recognize the potential for error or be able to check the plausibility of the recommendations made by LLM-based applications. For these reasons, careful evaluation of such systems is essential to ensure the safety and health of users.

The European Union AI Act is a comprehensive legal framework for AI systems [8]. It establishes a risk-based approach by assigning systems to risk classes, namely, unacceptable risk, high risk, limited risk, and minimal risk, and imposes corresponding requirements, with most obligations for providers of high-risk systems. Under the AI Act, systems are classified as high risk if their failure or malfunction could pose significant risks to an individual’s health, safety, or fundamental rights. Consequently, numerous applications within the medical domain are considered high risk and are therefore subject to stringent requirements. While the AI Act demands evaluation on transparency, human oversight, accuracy, robustness, and cybersecurity (chapter 3, articles 13-15), it does not specify how these should be measured or which standards should be applied.

Previous studies have investigated evaluation methods of LLMs in clinical medicine and show that the metrics most examined are accuracy and consistency [9,10]. Benchmarks to evaluate the natural language understanding and generation of LLMs are Bilingual Evaluation Understudy [11], Recall-Oriented Understudy for Gisting Evaluation [12], Metric for Evaluation of Translation With Explicit Ordering, and BERTScore [13]. To test LLMs’ ability to apply medical knowledge, the MedQA dataset [14] is commonly used. It uses a questionnaire adapted from a standardized, multistage examination that physicians must pass to obtain a medical license in the United States [15,16]. Other frequently used benchmarks to test medical knowledge are MultiMedQA [17], PubMedQA [18], and MedCaseReasoning [19].

Several publications point out the need for standardized evaluation frameworks to address clinical needs [9,20,21]. Others propose a systematic human approach for evaluating LLMs that support clinical tasks [22] or frameworks to evaluate the clinical skills of LLM-based applications [23-26]. Most of this research focuses on the evaluation of clinical applications and use by health care professionals.

### Objectives

This study aims to identify evaluation approaches of LLM-based applications used by nonprofessionals. The approaches are assigned to dimensions, sorted according to their metrics, and listed according to the measuring instruments used. The following research questions are addressed:

1. What are the quality dimensions of current evaluation approaches for LLM-based applications for nonprofessional users?
2. How are these dimensions operationalized in terms of metrics and measurement instruments?

## Methods

### Overview

The Joana Briggs Institute methodology [27] will be used as the overarching approach for conducting and documenting this scoping review. For the protocol, the PRISMA-P (Preferred Reporting Items for Systematic Reviews and Meta-Analyses Protocols) [28] guidelines were used. A completed PRISMA-P checklist for this protocol can be found in Multimedia Appendix 1. The completed scoping review will be reported according to the PRISMA-ScR (Preferred Reporting Items for Systematic Reviews and Meta-Analyses extension for Scoping Reviews) [29] guidelines.

### Protocol and Registration

This protocol is authored by the research team and underwent peer review as a research protocol by JMIR Publications in July 2026 [30]. This protocol was registered retrospectively with the Open Science Framework on July 24, 2026, after search and screening was substantially underway. Registration was initiated once the team determined that formal Open Science Framework registration would strengthen the transparency and reproducibility of the review. No deviations from the registered protocol occurred during the search and screening phase of the review. The codebook for classifying evaluation dimensions remains subject to iterative refinement during data extraction and synthesis. Any substantive changes will be reported in the final manuscript.

### Eligibility Criteria

The population, concept, and context scheme [31] was used to define the inclusion criteria. Additional criteria concerning the classification of source types were introduced. Exclusion criteria were defined to complement the inclusion criteria and enhance clarity in the screening process. Table 1 shows the inclusion and exclusion criteria and their mapping to the population, concept, and context framework. The restricted inclusion to publications from the last 5 years (from January 2021 to January 2026) was chosen due to the fast-paced innovations in this field of research. This time frame includes the launch of ChatGPT and other important publicly available chatbots. The language restrictions to English and German were chosen due to resource constraints in personnel and language competence of the authors.

**Table 1:** Inclusion and exclusion criteria and their reference to the population, concept, and context schema.

	Inclusion criteria	Exclusion criteria
General	Published in 2021-2025 English or German language Primary studies with own data collection Full text available	Published before 2021 Other languages Secondary work without own data collection; comments No full-text access
Population	Explicitly used by or intended to be used by individuals without formal training in health care professions (eg, patients and relatives), including systems designed for dual use by professional health care providers and laypersons; this includes direct prompting from the patient perspective (“I have pain in...”) and indirect questioning (“Supply important patient information for...”)	Exclusive use by medical personnel or other professional health care providers or no specific description of the target audience
Concept	Technology: LLM^a^-based systems, transformer language models, and RAG^b^; this comprises both proprietary systems that interface with LLMs via APIs and publicly accessible LLM chatbots, including specialized medical chatbots and general-purpose chatbots that are queried for medical purposes Evaluation: evaluation mechanisms described	Exclusively rule-based chatbots or other systems without the use of LLMs No evaluation mechanisms; purely architectural descriptions
Context	Health care, prevention, health navigation, patient education, shared decision-making, self-diagnosis, symptom checking, and self-triage	No reference to health care or an exclusively professional context, clinical workflows, clinical research, triage, and clinical decision-making without active involvement of patients

^a^LLM: large language model.

^b^RAG: retrieval-augmented generation.

### Information Sources

The following databases were selected to comprehensively cover the interdisciplinary scope of the review. Medical and health science databases (PubMed, CINAHL, and PsycInfo) were consulted to capture literature on digital health, patient engagement, and measurement instruments. The IEEE Xplore database specializes in technology and computer science and was included to ensure coverage of research on LLMs, system architectures, and evaluation methodologies. The use of generative AI for literature research was transparently documented (model, version, prompts, and date) and made reproducible.

Publications in both German and English were considered. To find further relevant sources, a backward citation search for included conference papers was performed after completion of screening, in which all references of the included sources were screened using the same inclusion criteria. By reviewing the sources of the included papers as part of the full-text screening process, conferences referenced in those sources were also included.

### Search Strategy

In the first step, an initial search was conducted on December 1, 2025, in PubMed and IEEE Xplore using the search string “Large Language Model Evaluation health care,” yielding 1300 and 119 results, respectively. The first 50 results from each database were screened. Titles, abstracts, and index terms of these potentially relevant publications were analyzed to identify additional search terms. The resulting concepts and synonyms are summarized in Table 2.

**Table 2:** Concepts and their related terms.

Concept	Related terms
Evaluation	“Assessment,” “validation,” “appraisal,” “effectiveness,” “usability,” “performance,” “safety,” “acceptability,” “user experience,” “evidence,” “objective metrics,” and “review methods”
Large language model	“LLM,” “chatbot,” “GPT,” “ChatGPT,” “generative AI,” “genAI,” and “foundation models”
Health care	“Health care,” “clinical care,” “medical care,” “digital health,” “patient care,” “patient information,” “patient education,” “patient management,” “patient assistance,” “personal health,” and “medical”

The search terms were combined to construct the search strings using the “OR” operator within individual concepts and the “AND” operator between different concepts. The strings were designed to search for the specified terms in titles and abstracts and adapted as required for each database. Textbox 1 provides an example of the PubMed search string, where “[tiab]” indicates a title and abstract search. For this search string, MeSH terms [32] were used to capture established concepts, and free-text terms were used to include emerging topics and topics that were not covered by the MeSH library. The complete search strings for all databases are provided in Multimedia Appendix 2.

**PubMed search string.**

( “Evaluation Studies as Topic”[Mesh] OR Assessment[tiab] OR Validation[tiab] OR Appraisal[tiab] OR Effectiveness[tiab] OR Usability[tiab] OR Performance[tiab] OR “Safety” [Mesh] OR “Patient Acceptance of Health Care” [Mesh] OR “User Experience”[tiab] OR Evidence[tiab] OR “Objective Metrics”[tiab] OR “Review Methods”[tiab]) AND (“Large Language Models”[mesh] OR LLM[tiab] OR Chatbot*[tiab] OR GPT[tiab] OR ChatGPT[tiab] OR “Generative Artificial Intelligence”[Mesh] OR “Foundation Models”[tiab]) AND ( “Delivery of Health Care”[Mesh] OR “Clinical Care”[tiab] OR “Medical Care”[tiab] OR “Digital Health”[Mesh] OR “Patient Care”[Mesh] OR “Patient Information” [tiab] OR “Patient Education as Topic” [Mesh] OR “Patient Care Management”[Mesh] OR “Patient Assistance”[tiab] OR “Personal Health Services”[Mesh] OR “Medical”)

### Screening

**Figure 1:**
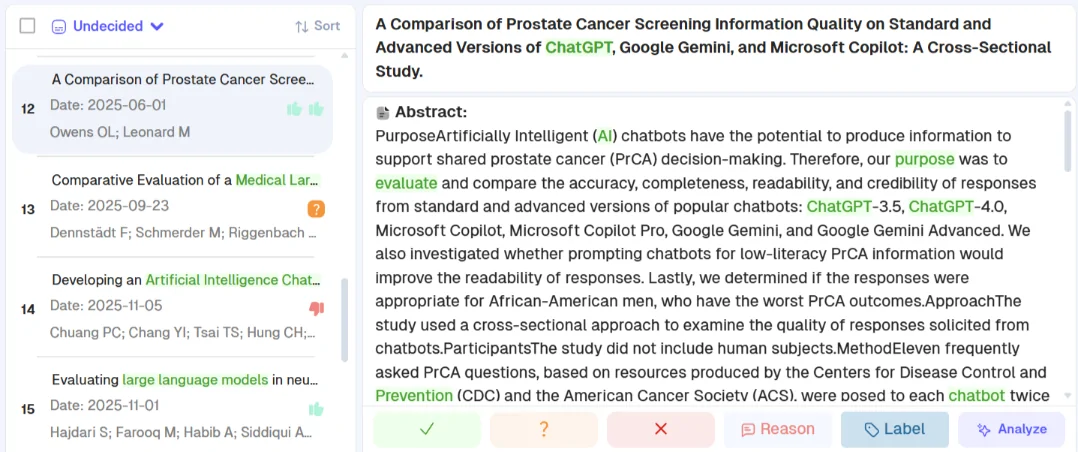
Screenshot of screening software Rayyan showing the highlighting of keywords and suggestions for decisions.

To achieve high interrater reliability, a set of 50 publications was screened by all reviewers, and the results were discussed to resolve discrepancies. Studies were selected based on the above-mentioned inclusion criteria and were reviewed in 2 stages. In the first stage, titles and abstracts were reviewed, followed by the full texts. Six reviewers screened the titles, abstracts, and full texts of the publications in pairs. This process was conducted in a blinded manner, meaning that reviewers could not see the evaluations provided by others while screening the publications. In the event of disagreements, a third reviewer was consulted. Remaining disagreements regarding study selection and data extraction were resolved through discussion and consensus with the review team. The web-based tool Rayyan (Rayyan Systems Inc) [33] was used to screen publications and manage resources. Rayyan provides AI-assisted support, which in this study was used to identify and remove duplicates and highlight key terms within publications. Additionally, Rayyan makes suggestions for inclusion or exclusion. The software interface with the aforementioned functions is shown in Figure 1.

### Data Charting

Included publications will be analyzed, and data will be extracted in tabular form. A template with PRISMA-ScR–compliant data items adapted from a methodological guide for scoping reviews [31] will be used as an overall starting framework. The selection of data fields is preliminary and will be iteratively refined by the review team if additional relevant information dimensions are identified in the included publications. Data extraction and charting will be performed by 1 reviewer. To ensure quality, a random sample of 20 publications will be independently charted by a second reviewer for calibration. Interrater reliability will be assessed using the Cohen κ, with a prespecified threshold above 0.61 required among reviewers in this subsample. If this threshold is not met, reviewers will undergo a recalibration process where discrepant ratings will be discussed and category definitions will be refined. A new sample of 20 publications will be independently rerated, and the charting process and data items will be iteratively refined until the threshold is achieved.

### Data Items

The preliminary data items are (1) reference (title, author, journal, year, and page), (2) study type, (3) population, (4) context (health care task), (5) type of technology used (LLM name and version), (6) evaluation dimension, (7) metric, (8) measuring instrument, and (9) use of reporting frameworks (such as CONSORT-AI [Consolidated Standards of Reporting Trials–AI] [34]).

### Coding

The data charting step will be followed by the coding of quality dimensions, metrics, and measurement instruments using a deductive-inductive coding scheme with iterative refinement of the coding book based on the principles of qualitative content analysis by Mayring [35]. While the evaluation items will be previously extracted verbatim from the publications, they will be subsequently coded to superordinate dimensions. The coding and calibration process will follow a stepwise approach with iterative refinement of the codebook. The initial codebook for evaluation dimensions uses the categories suggested by the AI Risk Management Framework (RMF) [36] for AI risks and trustworthiness. The AI RMF suggests seven dimensions of risks and trustworthiness: (1) validity and reliability, (2) safety, (3) security and resilience, (4) accountability and transparency, (5) explainability and interpretability, (6) privacy enhancement, and (7) fairness.

In the first step of pilot coding, these dimensions will be used as categories and charted as present or not present for each publication (deductive step). To chart the metrics used for each quality dimension, the exact wording in the publication will be used (eg, “accuracy” for the first dimension). The measuring instrument will describe the actual evaluation method used (eg, “5-point ordinal scale”) and the evaluating instance (eg, “medical professional” or “software”). An example is provided in Table 3. Metrics reported in studies that cannot be mapped to the predefined categories provided by the AI RMF will be charted as possible emergent categories. The pilot coding will include a random set of 20 publications, which will be double screened by 2 independent coders. Following the pilot phase, coders will compare results and discuss disagreements and emergent dimensions. Newly identified categories will be added to the codebook if they represent a distinct concept not encompassed by any existing dimension, and both raters will agree on whether the emergent category is sufficiently supported by data. Subsequently, to ensure reliability, the codebook will undergo iterative calibration rounds. In each calibration round, both coders will independently code a new random set of 20 publications. Intercoder reliability will be measured using the Cohen κ. Disagreements will be resolved through discussion, leading to further refinement of the codebook. The calibration process will continue until the Cohen κ reaches 0.61, reflecting substantial agreement between coders. Once calibration achieves the reliability threshold, the finalized codebook will be used for the primary coding of the remaining publications. To enhance efficiency, coding will be performed by a single coder following the established guidelines in the codebook. A random subset of 10% will undergo additional independent coding by the second rater to confirm reliability during main coding. The coding process will implement transparency via systematic documentation of all adjustments to dimensions, inclusions of new categories, and discussions.

**Table 3:** Example of data charting of dimensions, metrics, and measuring instruments.

	Dimension	Metric	Measuring instrument
Validity and reliability	Yes	Accuracy Reliability	Accuracy: 5-point ordinal scale Reliability: 3-point ordinal scale Both rated by 2 medical professionals
Safety	No	—^a^	—
Explainability and interpretability	Yes	Readability	Flesch-Kincaid reading grade level Automated via web tool

^a^Not applicable

### Critical Appraisal

There is no formal quality assessment planned (Joana Briggs Institute compliant) as the goal is to map the evidence field.

### Synthesis of Results

A PRISMA (Preferred Reporting Items for Systematic Reviews and Meta-Analyses) flowchart will be generated to illustrate the various reasons for excluding publications. A template from PRISMA will be used for this purpose and will be available in the publication of the scoping review results. Charted data will be managed according to the a priori–defined data items. Data will be aggregated across studies and summarized using descriptive statistics and structured narrative synthesis. A deductive-inductive coding scheme will be used to determine the quality dimensions.

The results will be presented in tables, figures, and a summary description to illustrate the scope and characteristics of the evaluation mechanisms used. The review will be reported in accordance with PRISMA-ScR guidelines.

## Results

**Figure 2:**
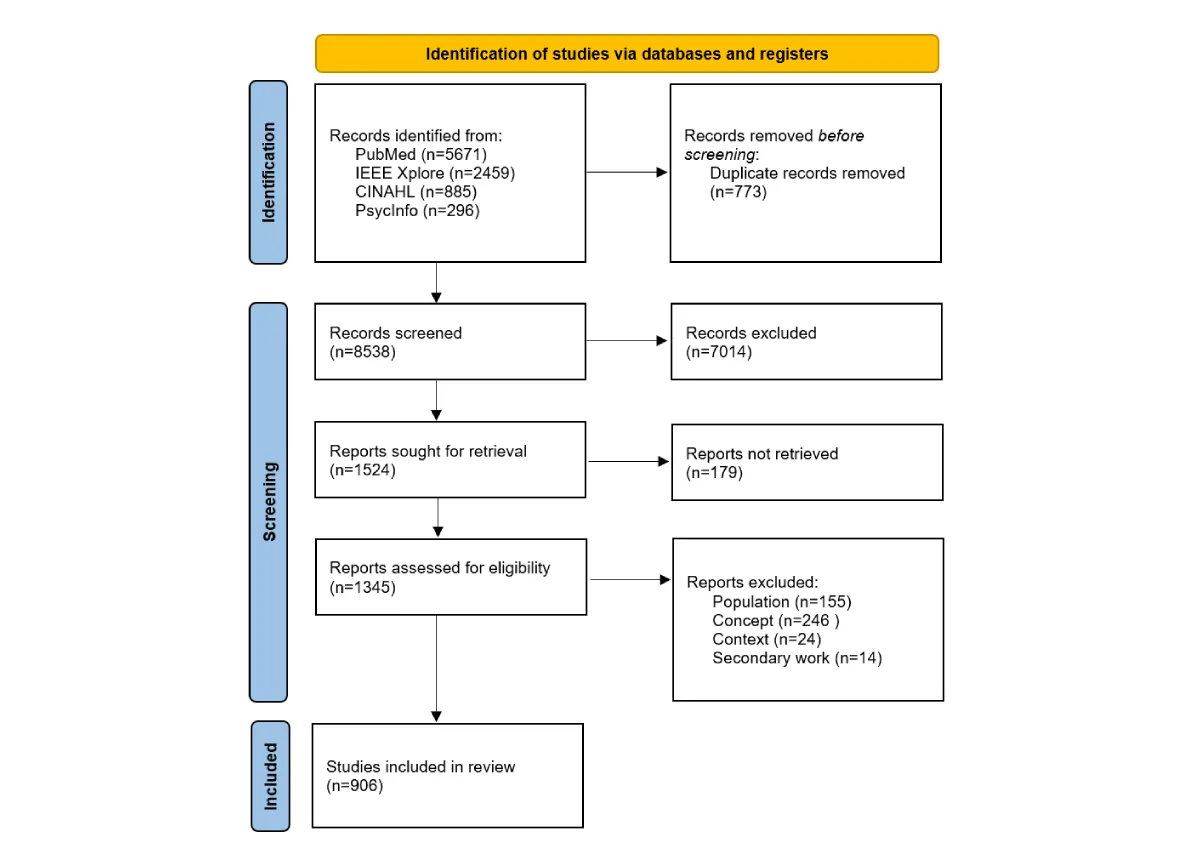
PRISMA (Preferred Reporting Items for Systematic Reviews and Meta-Analyses) flowchart of records in the phases of identification, screening, and inclusion (template from Page et al [37]).

In January 2026, a steering committee of 6 researchers was established to carry out the review. A search performed on January 20, 2026, on all databases yielded 9311 results, including 60.9% (5671/9311) results from PubMed, 26.4% (2459/9311) results from IEEE Xplore, 9.5% (885/9311) results from CINAHL, and 3.2% (296/9311) results from PsycInfo. Of these 9311 publications, after removing 8.3% (773/9311) duplicates, 91.7% (8538/9311) remained for screening. As of July 2026, after screening, 1524 publications remained for retrieval, from which 88.3% (1345/1524) records were retrieved. In the following step of full-text screening, of the 1345 retrieved records, 11.5% (155/1345) were excluded due to nonmatching populations (see details in the Eligibility Criteria section), 18.3% (246/1345) were excluded due to nonmatching concepts (other technology or no evaluation described), 1.8% (24/1345) were excluded due to nonmatching contexts, and 1% (14/1345) were excluded due to being secondary work. This resulted in 906 publications remaining for data charting. This count is provisional pending second-reviewer verification in accordance with the protocol. Data collection, coding and synthesis of the results had not been started as of August 5, 2026. The expected start for these 2 phases is the middle of August 2026. The PRISMA flowchart in Figure 2 [37] shows the number of studies that were identified, screened, and included.

We expect the final full-text screening, data extraction, coding, and synthesis results for the fourth quarter of 2026.

## Discussion

### Anticipated Findings

The expected outcome of this study is primarily an overview of mechanisms for evaluating LLM systems used in health care by nonprofessionals. In this process, certain quality dimensions, most notably accuracy, are likely to emerge as prominent, having been examined across numerous studies, whereas other dimensions are investigated in only a limited subset of the literature. We expect to identify a set of dimensions that are evaluated in current publications and the degree of alignment with current definition categories. The outcome includes metrics used to describe the specific matter evaluated in each dimension. We also expect a comprehensive overview of the measuring instruments used to evaluate each metric. We anticipate finding dominant or underrepresented dimensions and metrics and identify common operationalizations for measurements.

### Comparison With Prior Work

This study builds on previous work and extends existing approaches to categorizing and mapping evaluation mechanisms by incorporating the specific perspective of use by nonprofessionals. Prior work has largely emphasized systems facing health care professionals, whereas our focus allows for insights into how quality dimensions are operationalized by nonprofessional users for health care queries.

### Dissemination Plan

The findings of this review will be disseminated through multiple channels to different stakeholder groups. Results will be submitted to a peer-reviewed journal and as international conference papers to connect with the scientific community. A preprint of this research protocol and the subsequent research paper will be made available online. To reach practitioners and experts, open workshops will be held to discuss the practical applicability and relevance of an evaluation guideline for LLM chatbots in health care. The research findings will be adapted for the general public in summaries that are accessible to nonexperts and published by the associated organizations of the authors on social media platforms.

### Limitations

A general limitation of scoping reviews is their lack of strict quality assessment of sources. Another limitation is the exclusion of non–English- or non–German-language publications; this may overlook contributions from research groups working in other languages. Given the prevalence of publication bias in scientific research, the predominance of positive results in this study may likewise skew the assessment of the actual situation. The absence of a quality assessment limits the derivation of efficacy judgments. The results will serve to map the evidence and generate hypotheses.

Furthermore, the rapid evolution of LLMs implies that the findings may become outdated by the time of publication, particularly when the publication process involves lengthy peer review.

### Conclusions

The scoping review will provide a comprehensive overview of evaluation methods used in LLM applications for nonprofessionals in health care. It will show the range of evaluation methods and explain quality dimensions with their operationalization mechanisms applied in scientific work. It will identify research gaps and provide directional guidance for further research and development in the field of quality assurance of LLM applications for nonprofessional users.

## Appendix

Multimedia Appendix 1 Complete list of final search strings for all databases.

Multimedia Appendix 2 PRISMA-P checklist.

## Abbreviations

CONSORT-AI: Consolidated Standards of Reporting Trials–Artificial Intelligence
LLM: large language model
PRISMA: Preferred Reporting Items for Systematic Reviews and Meta-Analyses
PRISMA-P: Preferred Reporting Items for Systematic Reviews and Meta-Analyses Protocols
PRISMA-ScR: Preferred Reporting Items for Systematic Reviews and Meta-Analyses extension for Scoping Reviews
RAG: retrieval-augmented generation
RMF: Risk Management Framework
