# Supplementary material for: Methods of Evaluating Large Language Model–Based Health Care Applications Used by Nonprofessionals: Protocol for a Scoping Review
Source: JMIR Res Protoc. 2026 Aug 11;15:e93509. doi: 10.2196/93509 (PMC13507720; doi:10.2196/93509)
Supplement: Supplementary file 1 [file resprot_v15i1e93509_app1.pdf]

PRISMA-P (Preferred Reporting Items for Systematic review and Meta-Analysis Protocols) 2015 Checklist

Recommended items to address in a systematic review protocol*

Section & Topic	Item No.	Checklist Item
**ADMINISTRATIVE INFO**		
**Title**		
Identification	1a	Evaluation Methods for LLM-based Applications for Non-professional Users in Healthcare: A Scoping Review Protocol
Update	1b	This protocol is no update of a previous systematic review
**Registration**		
Registration	2	Retrospectively registered on OSF (July 24, 2026), DOI: https://doi.org/10.17605/OSF.IO/R2UEM. No deviations during search/screening; future substantive codebook changes to be reported (Protocol and Registration).
**Authors**		
Contact	3a	Maren Keuchel, Witten/Herdecke University, maren.keuchel@uni-wh.de; Pinar Bisgin, Fraunhofer ISST, pinar.bisgin@isst.fraunhofer.de; Tom Strube, Fraunhofer ISST, tom.strube@isst.fraunhofer.de; Niklas Tschorn, niklas.tschorn@isst.fraunhofer.de; Leoni Weltermann, Fraunhofer ISST, leoni.weltermann@isst.fraunhofer.de; Sven Meister, Witten/Herdecke University, sven.meister@uni-wh.de
Contributions	3b	MK conceptualization, writing and original draft,<br>PB, TS, LW, NT writing, review and editing<br>SM supervision
**Amendments**		
Amendments	4	Registration occurred after search/screening was underway; no deviations in those phases. Codebook may be iteratively refined during extraction/synthesis; any substantive changes will be reported in the final manuscript
**Support**		
Sources	5a	No financial support or grants received
Sponsor	5b	none
Role of Sponsor or Funder	5c	not applicable
**INTRODUCTION**		
**Rationale**		
Rationale	6	Described in Section Background
**Objectives**		
Objectives	7	Described in Section Objectives
**METHODS**		
**Eligibility Criteria**		
Eligibility criteria	8	PCC framework, described in section eligibility criteria

**Information Sources**		
Information sources	9	Described in Section Information source
**Search Strategy**		
Search strategy	10	Described in Section seach strategy
**Study Records**		
Data management	11a	Screening and management in Rayyan, described in Section Screening and Section Data Charting
Selection process	11b	Described in Section Screening
Data collection process	11c	Described in Section Data Charting and Section Coding
**Data Items**		
Data items	12	Described and listed in Section Data Items
**Outcomes and Prioritization**		
Outcomes and prioritization	13	Mapping of evaluation dimensions, metrics, and instruments. Described in Methods overview
**Risk of Bias**		
Risk of bias in individual studies	14	Not applicable for scoping reviews, no formal quality assessment planned
**Data Synthesis**		
Data synthesis	15a	Not applicable, no meta-analysis planned
	15b	Descriptive statistics and structured narrative synthesis, deductive–inductive coding to determine dimensions. Described in Synthesis of Results and Section Coding
	15c	Iterative codebook calibration, identification of emergent categories beyond AI RMF. Described in Section Coding
	15d	Narrative and tabular summaries, PRISMA flow diagram for study selection. Described in Synthesis of the Result
**Meta-bias(es)**		
Meta-bias(es)	16	Not applicable for scoping reviews
**Confidence in Evidence**		
Confidence in cumulative evidence	17	Not applicable for scoping reviews

*It is strongly recommended that this checklist be read in conjunction with the PRISMA-P Explanation and Elaboration for important clarification on the items. Amendments to a review protocol should be tracked and dated. The copyright for PRISMA-P (including checklist) is held by the PRISMA-P Group and is distributed under a Creative Commons Attribution Licence 4.0.

From: *Shamseer L, Moher D, Clarke M, Ghersi D, Liberati A, Petticrew M, Shekelle P, Stewart L, PRISMA-P Group. Preferred reporting items for systematic review and meta-analysis protocols (PRISMA-P) 2015: elaboration and explanation. BMJ. 2015 Jan 2;349(jan02 1):g7647.*
