# Supplementary material for: Methods of Evaluating Large Language Model–Based Health Care Applications Used by Nonprofessionals: Protocol for a Scoping Review
Source: JMIR Res Protoc. 2026 Aug 11;15:e93509. doi: 10.2196/93509 (PMC13507720; doi:10.2196/93509)
Supplement: Supplementary file 2 [file resprot_v15i1e93509_app2.pdf]

Search Strings

PubMed

("Evaluation Studies as Topic"[Mesh] OR Assessment[tiab] OR Validation[tiab] OR Appraisal[tiab]

OR Effectiveness[tiab] OR Usability[tiab] OR Performance[tiab]

OR "Safety"[Mesh] OR "Patient Acceptance of Health Care"[Mesh] OR "User Experience"[tiab]

OR Evidence[tiab] OR "Objective Metrics"[tiab] OR "Review Methods"[tiab])

AND

(„Large Language Models"[mesh] OR LLM[tiab] OR Chatbot*[tiab] OR GPT[tiab]

OR ChatGPT[tiab] OR "Generative Artificial Intelligence"[Mesh] OR "Foundation Models"[tiab])

AND

("Delivery of Health Care"[Mesh] OR "Clinical Care"[tiab] OR "Medical Care"[tiab]

OR"Digital Health"[Mesh] OR "Patient Care"[Mesh] OR "Patient Information"[tiab]

OR"Patient Education as Topic"[Mesh] OR"Patient Care Management"[Mesh] OR "Patient Assistance"[tiab] OR "Personal Health Services"[Mesh] OR "Medical")

CINAHL & PsychInfo

(TI(Evaluation) OR AB(Evaluation) OR TI(Assessment) OR AB(Assessment)

OR TI(Validation) OR AB(Validation) OR TI(Appraisal) OR AB(Appraisal)

OR TI(Effectiveness) OR AB(Effectiveness) OR TI(Usability) OR AB(Usability)

OR TI(Performance) OR AB(Performance) OR TI(Safety) OR AB(Safety)

OR TI(Acceptability) OR AB(Acceptability) OR TI("User Experience") OR AB("User Experience")

OR TI(Evidence) OR AB(Evidence) OR TI("Objective Metrics") OR AB("Objective Metrics")

OR TI("Review Methods") OR AB("Review Methods")

)

AND

(TI("Large Language Model") OR AB("Large Language Model") OR TI(LLM) OR AB(LLM)

OR TI(Chatbot) OR AB(Chatbot) OR TI(GPT) OR AB(GPT) OR TI(ChatGPT) OR AB(ChatGPT)

OR TI("Generative AI") OR AB("Generative AI") OR TI(GenAI) OR AB(GenAI)

OR TI("Foundation Models") OR AB("Foundation Models")

)

AND

(TI("Health Care") OR AB("Health Care") OR TI(Healthcare) OR AB(Healthcare)

OR TI("Clinical Care") OR AB("Clinical Care") OR TI("Medical Care") OR AB("Medical Care")

OR TI("Digital Health") OR AB("Digital Health") OR TI("Patient Care") OR AB("Patient Care")

OR TI("Patient Information") OR AB("Patient Information") OR TI("Patient Education") OR AB("Patient Education")

OR TI("Patient Management") OR AB("Patient Management") OR TI("Patient Assistance") OR AB("Patient Assistance")

OR TI("Personal Health") OR AB("Personal Health") OR TI(Medical) OR AB(Medical)

)

IEEE Xplore

(("Evaluation" OR "Assessment" OR "Validation" OR "Appraisal"

OR "Effectiveness" OR "Usability" OR "Performance"

OR "Safety" OR "Acceptability" OR "User Experience"

OR "Evidence" OR "Objective Metrics" OR "Review Methods")

AND

(

"Large Language Model" OR LLM OR Chatbot OR GPT

OR ChatGPT OR "Generative AI" OR GenAI OR "Foundation Models"

)

AND

(

"Health Care" OR Healthcare OR "Clinical Care" OR "Medical Care"

OR "Digital Health" OR "Patient Care" OR "Patient Information"

OR "Patient Education" OR "Patient Management" OR "Patient Assistance"

OR "Personal Health" OR “Medical”

))
